# Transposon insertion sequencing reveals T4SS as the major genetic trait for conjugation transfer of multi-drug resistance pEIB202 from *Edwardsiella*

**DOI:** 10.1186/s12866-017-1013-7

**Published:** 2017-05-12

**Authors:** Yang Liu, Yanan Gao, Xiaohong Liu, Qin Liu, Yuanxing Zhang, Qiyao Wang, Jingfan Xiao

**Affiliations:** 1 State Key Laboratory of Bioreactor Engineering, East China University of Science and Technology, Shanghai, 200237 China; 2 Shanghai Engineering Research Center of Maricultured Animal Vaccines, Shanghai, China; 3 Shanghai Collaborative Innovation Center for Biomanufacturing, 130 Meilong Road, Shanghai, 200237 China

**Keywords:** Plasmid conjugation, *Edwardsiella Piscicida*, Tis, T4SS, RNA-seq, EsrB

## Abstract

**Background:**

Conjugation is a major type of horizontal transmission of genes that involves transfer of a plasmid into a recipient using specific conjugation machinery, which results in an extended spectrum of bacterial antibiotics resistance. However, there is inadequate knowledge about the regulator and mechanisms that control the conjugation processes, especially in an aquaculture environment where a cocktail of antibiotics may be present. Here, we investigated these with pEIB202, a typical multi-drug resistant IncP plasmid encoding tetracycline, streptomycin, sulfonamide and chloramphenicol resistance in fish pathogen *Edwardsiella piscicida* strain EIB202.

**Results:**

We used transposon insertion sequencing (TIS) to identify genes that are responsible for conjugation transfer of pEIB202. All ten of the plasmid-borne type IV secretion system (T4SS) genes and a putative lipoprotein p007 were identified to play an important role in pEIB202 horizontal transfer. Antibiotics appear to modulate conjugation frequencies by repressing T4SS gene expression. In addition, we identified *topA* gene, which encodes topoisomerase I, as an inhibitor of pEIB202 transfer. Furthermore, the RNA-seq analysis of the response regulator EsrB encoded on the chromosome also revealed its essential role in facilitating the conjugation by upregulating the T4SS genes.

**Conclusions:**

Collectively, our screens unraveled the genetic basis of the conjugation transfer of pEIB202 and the influence of horizontally acquired EsrB on this process. Our results will improve the understanding of the mechanism of plasmid conjugation processes that facilitate dissemination of antibiotic resistance especially in aquaculture industries.

## Background

Antibiotic resistance (ABR) raises global issues regarding to multidrug-resistant and overuse of antibiotics [1]. It has been established that the spread of ABR and virulence is often mediated by mobile genetic elements including insertion sequence, transposons, integrons, bacteriophage, genomic island (such as pathogenicity island), plasmids and combinations of these elements [2–4]. Plasmids and other mobile DNA elements can be more easily transferred than chromosomal DNA between genera, phyla and even major domains by a mechanism known as conjugation [5–7]. Direct evidence has established that tetracycline resistance-encoding plasmids (R plasmid) disseminate between different *Aeromonas* species and *Escherichia coli* in various environments [8]. Horizontal gene transfer (HGT) is also important for genome plasticity. It has been reported that approximately 3/4 of genes in the bacterial genomes have been acquired by HGT [9], which is essential for bacteria to adapt to various growth niches. Though there are several reports about the genetic screen for bacterial plasmid conjugation, the knowledge about the detailed processes and genetic basis that control the transfer of the plasmid to another host cell is still lacking.

Bacteria utilize secretion systems to transport numerous substrates across cellular membranes, mediating their virulence and survival. The type IV secretion system T4SS is unique and present in many pathogens to mediate both genetic exchange and the delivery of effector proteins to target eukaryotic cells [10]. T4SS in *Agrobacterium tumefaciens* delivers oncogenic nucleoprotein particles into plant cells, resulting in the development of crown-gall tumors [11]. *A. tumefaciens* T4SS is composed of 12 proteins, VirB1 ~ 11 and VirD4, while VirB proteins can be grouped into three classes: the putative channel components (VirB6 – VirB10); the energy components (the nucleoside triphosphatases VirB4 and VirB11); and the pilus-associated components (VirB2, and possibly VirB3 and VirB5) [12]. The machines assembled from VirB homologs are proposed as type IVA (T4AS) and are widespread to mediate the conjugative transfer of plasmids and thus promote dissemination of multiple-antibiotic resistance [13].

*Edwardsiella piscicida* is a bacterial pathogen causing edwardsiellosis in over 20 piscine species such as flatfish, eel and tilapia, resulting in huge economic losses in worldwide aquaculture industries [14]. *E. piscicida* is a facultative intracellular pathogen and develops the ability to resist killing by professional phagocytes and colonize and replicate in macrophages [15–17]. In our previous study, the genome of a typical highly virulent *E. piscicida* strain EIB202 was published [18]. The horizontally acquired two-component system EsrA-EsrB has been established to be essential for its pathogenesis mediated by type III and VI secretion systems (T3SS and T6SS) [18–20]. A plasmid (pEIB202) of 43,703 bp was identified from the assembled sequence [18]. Six genes in the sequence of pEIB202 were identified to be involved with ABR, providing genetic properties for multi-drug resistance in EIB202, including *tetA* and *tetR* for tetracycline, *strA* and *strB* for streptomycin, *sulII* for sulfonamide, and *catA3* for chloramphenicol resistance. Genetic analysis suggested that the chloramphenicol resistance might be recently acquired by the plasmid [18]. In addition, this plasmid encoding an incomplete set of T4AS proteins (VirB2, -B4, -B5, -B6, -B8, -B9, -B10, -B11, -D2, and -D4) [18]. It is unknown whether these T4AS proteins involved in the acquisition of the plasmid by the bacterium.

In this study, we identified that the self-transmissible pEIB202 is not involved in virulence and colonization of *E. piscicida* at least in the zebrafish model we used in this study. We used transposon insertion sequencing (TIS) technology to investigate genetic basis of pEIB202 on conjugation transfer of pEIB202, and identified all of the T4SS related proteins on pEIB202 and putative lipoproteins as horizontal transfer enhancer, and the TopA as the related inhibitor. Intriguingly, response regulator EsrB encoded in the chromosome was found to facilitate the conjugation through inducing the expression of genes associated with antibiotics resistance and T4SS. Our data unraveled the genetic basis of the conjugation transfer of pEIB202 and the influence of horizontally acquired EsrB on the plasmid transfer efficiency.

## Methods

Bacterial strains, plasmids and culture conditions. Bacterial strains and plasmids used in this work were described in Table 1, respectively. *E. piscicida* strains were grown at 30 °C in tryptic soy broth (TSB) or Luria broth (LB). *E. coli* strains were cultured in LB at 37 °C. DH5α *λpir* were used for plasmid harvest, and SM10 *λpir* was used plasmid conjugation. Antibiotics were added to the following final concentrations: gentamicin (Gm, 25 μg/ml), polymyxin B (Col, 10 μg/ml), kanamycin (Km, 50 μg/ml), and carbenicillin (Carb, 100 μg/ml), chloramphenicol (Cm, 34 μg/ml), tetracycline (Tet, 12.5 μg/ml) and streptomycin (Str, 100 μg/ml). For the tests of the effect of antibiotics on conjugation frequency, the following sublethal concentrations (1/4 of minimum inhibition concentration (MIC)) of antibiotics were used: Tet, 0.312 μg/ml; Cm, 0.437 μg/ml, Str: 0.575 μg/ml.

**Table 1:** Strains and plasmids used in this study

Strain or plasmid	Characteristics^a^	Reference
*E. piscicida*		
EIB202	Wild type (CCTC No. M 208068) carrying pEIB202, Col^r^, Cm^r^, Str^r^	[33]
EIB202-*lacZ* ^+^	EIB202, insert a *lacZ* in *glmS*, Col^r^, Str^r^	Lab collection
EIB202 ∆P	EIB202, pEIB202 cured, Col^r^	[41]
∆*esrB*	EIB202, in-frame deletion of *esrB*, Col^r^, Cm^r^, Str^r^	[20]
∆*topA*	EIB202, in-frame deletion of *topA*, Col^r^, Cm^r^, Str^r^	This study
∆*p007*	EIB202, in-frame deletion of p007, Col^r^, Cm^r^, Str^r^	This study
∆*virB9*	EIB202, in-frame deletion of *virB9*, Col^r^, Cm^r^, Str^r^	This study
*esrB* ^*+*^	EIB202, ∆*esrB*, containing pAK*gfp*1::*flag*-*esrB*, Col^r^, Carb^r^	Lab collection
*topA* ^*+*^	EIB202, ∆*topA*, containing pAK*gfp*1::*topA*, Col^r^, Carb^r^	This study
*p007* ^*+*^	EIB202, ∆*p007*, containing pAK*gfp*1::*p007*, Col^r^, Carb^r^	This study
*virB9* ^*+*^	EIB202, ∆*virB9*, containing pAK*gfp*1::*virB9*, Col^r^, Carb^r^	This study
∆P::pNQ705K	EIB202 ∆P with pNQ705K inserted in downstream of *glms*, Col^r^, Km^r^	This study
*E. coli*		
DH5α *λpir*	Δ (*lacZYA-argF*) U169 (Φ80 *LacZ ΔM15*), *pir* dependent *R6K.*	Lab collection
SM10 *λpir*	*thi thr leu tonA lacY supE recA::RP4–2-Tc::Mu, pir* dependent *R6K*, Km^r^	Lab collection
*Vibrio harveyi*		
BB170	*Vibrio harveyi*, reporter strain, Carb^r^	Lab collection
*Vibrio alginolyticus*		
∆T6SS	*Vibrio alginolyticus*, T6SS genes were deleted, Carb^r^	Lab collection
Plasmid		
pMPR	Mariner transposon plasmid, *pir* dependent R6K, Gm^r^, Carb^r^	Lab collection
pAKgfp1	pBBRMCS4 with *gfpmut3a*	Lab collection
pNQ705	Suicide plasmid, *pir* dependent R6K, Cm^r^	[53]
pNQ705K	pNQ705 derivative with Km fragment inserted in *SalI* site, Cm^r^, Km^r^	This study
pNQ705K-*glmS*	pNQ705K derivative with *glmS* fragment inserted in *XbaI* site, Cm^r^, Km^r^	This study
pDM4	Suicide plasmid, *pir* dependent, R6K, *sacBR*, Cm^r^	[54]
pDMK	pDM4 derivative with Km fragment inserted in *SalI* site, Km^r^, Cm^r^	[25]
pD43B	pDMK derivative with *virB10* and *gyrB* fragment inserted in *XbaI* site, Km^r^, Cm^r^	This study
p34S-Km	Cloning vector, Km^r^	[55]

^a^Col^r^, polymyxin B resistance; Cm^r^, chloroamphenicol resistance; Km^r^, kanamycin resistance; Carb^r^, carbenicillin resistance; Gm^r^, gentamycin resistance

Quantitative real-time PCR (qRT-PCR). RNA samples were extracted by using the RNA isolation kit (Tiangen). RNase-free DNase I (Promega) was used to remove DNA contamination in the RNA sample. One microgram RNA was used as a template for first strand cDNA synthesis with the PrimeScript reverse transcriptase (TaKaRa). qRT-PCR analysis was performed in a total volume of 20 μl, containing 1 μl of diluted cDNA, 1 μl of each primer (10 mM stock) (Table 2), 10 μl of FastStart Universal SYBR Green Master (Roche) and 7 μl deionized water. qRT-PCR was performed with the 7500 RealTime PCR System (Applied Biosystems) under the following conditions: 95 °C for 10 min; 40 cycles at 95 °C for 15 s, and 60 °C for 1 min. Melting curve analysis of amplification products was performed at the end of each PCR to confirm that only one PCR product had been amplified. Relative quantification was performed using the comparative CT (2^-ΔΔCT^) method [21], with the housekeeping gene *gyrB* as an internal control.

**Table 2:** Primers used in this study

Oligonucleotides	Sequence
Glms-F	CCCCCCCGAGCTCAGGTTACCCGGATCTATGGAAATCGGCGTAGCGTCAACCAAG
Glms-R	CCCTCGAGTACGCGTCACTAGTGGGGCCCTTCGCGCTTTTATTCTACGGTAACCG
Glms-yz-F	AGAGATTGGCTACTTGGGATCGTTG
KmCS-F (SalI)	ACGCGTCGACATTGTGAGCGGATAACAATTTGTGG
KmCS-R (SalI)	ACGCGTCGACTAGATCCGGGTAACCTGAGCT
*evpP*-F	TCATCGCACATACAGAATAAACGCC
*evpP*-R	CCGTAACATTTCTTACAACACTGCG
*virB9*-P1	CCCCCCCGAGCTCAGGTTACCCGGATCTATGGCCACTGGTTTGTTGTAGGGCCAT
*virB9*-P2	CAAATTGCCATGGGCTGATGGCTGAGAACAGAGACGATCTGGA
*virB9*-P3	TGTTCTCAGCCATCAGCCCATGGCAATTTGTTGGACCGTT
*virB9*-P4	GAGTACGCGTCACTAGTGGGGCCCTTCTAGCGCGAGGGCTATCAGTGGGAAACCC
*virB9*-out-F	GCGCCCAGGCCGTCCGCTCGTTCAG
*virB9*-out-R	CCGCGTCGATAACAACACTGGCGTG
*virB9*-in-F	TTGACTCCCTCTAATTACTCGCTCA
*virB9*-in-R	GTCGGAAATCACATTTTCATCAAGC
p007-P1	CCCCCCCGAGCTCAGGTTACCCGGATCTATTGGCGCGGGTCGGTATATGCGGCAT
p007-P2	AGGAGAGTTCGAGTGGCTTGGTCATCCGAGGAATGGAGGC
p007-P3	CTCGGATGACCAAGCCACTCGAACTCTCCTTGATCAGTGT
p007-P4	GAGTACGCGTCACTAGTGGGGCCCTTCTAGTTTTTGAAAGCTGGCTAGGCATGGT
p007-out-F	CTGCGCTCCCCTGCCCTTTTCACCT
p007-out-R	TGCGCTTTCTCTCGTTGTGGCGTTC
*topA*-P1	CCCCCCCGAGCTCAGGTTACCCGGATCTATGGTCCAGGACCCAATCCACCCCTTC
*topA*-P2	TAGGGAGGACCAATGTGAGCTTGGCGGCAATCAAAGTTGT
*topA*-P3	TTGCCGCCAAGCTCACATTGGTCCTCCCTACCGTCAACCA
*topA*-P4	GAGTACGCGTCACTAGTGGGGCCCTTCTAGACCTCCAGTCGGTCGAGTTGAGCAA
*topA*-out-F	GGCCGCGATAATCAGGTCAACGATG
*topA*-out-R	CACCTGTGCGGCCCTGTCCGGGGCT
*topA*-in-F	ACCAAAAATCGTAACCCTTCTTGCC
*topA*-in-R	AGGGTGGGACGTGCAGGCCAGTGTC
pDMK-PF	AAAGCTCTCATCAACCGTGGC
pDMK-PR	TGCTCCAGTGGCTTCTGTTTC
*topA*-C-F	GATCCTCTAGATTTAAGAAGGAGATATACAATGAATCTAGTTATTGTTGA
*topA*-C-R	GATCCCCCGGGCTGCAGGAATTCGATATCATCAAATCTTTGGCTTGCCAC
p007-C-F	GATCCTCTAGATTTAAGAAGGAGATATACAGTGAACCTGAAAACACTAAG
p007-C-R	GATCCCCCGGGCTGCAGGAATTCGATATCATCAGTGATGGCCTCCATTCC
*virB9*-C-F	GATCCTCTAGATTTAAGAAGGAGATATACAATGATACGAGCAAAATCACT
*virB9*-C-R	GATCCCCCGGGCTGCAGGAATTCGATATCATCATTGACTCCCTCTAATTA
*virB9*-qRT-F	TCATGTTCGTGGTCGCATCA
*virB9*-qRT-R	CCATTTTGGCTTCTCCACGC
*virB4*-qRT-F	TGGGGGCCGTTTTGAGATAC
*virB4*-qRT-R	GCGGCAGCTTCAATAACCAG
*avtA*-qRT-F	TTACTCCGCAATCACCCGTC
*avtA*-qRT-R	CAGCTCACCGCATAGGGAG
*P021*-qRT-F	AAAGCCCCAAACCGTAAAGC
*P021*-qRT-R	ATGCGGGAATGGGTCAGTTT
*gyrB*-qRT-F	CCGATGATGGTACGGGTCTG
*gyrB*-qRT-R	GCTTTTCAGACAGGGCGTTC
*p003*-qRT-F	GCCGAAGCGTTCCCAAAAAT
*p003*-qRT-R	CCTGTGGAATCGCATCGAGA
pD43B-*p003*-F	GAGCTCAGGTTACCCGCATGCAAGATCTATATGATGGCTGAGAACAGAGACGATC
pD43B-*p003*-R	ATACGTATTTGACATTCAATCGGCTTTGAGGTCATATACC
pD43B-*gyrB*-F	CTCAAAGCCGATTGAATGTCAAATACGTATGACTCCTCAA
pD43B-*gyrB*-R	CCCTCGAGTACGCGTCACTAGTGGGGCCCTTTAAAAGTCCAGATTGGACGCTTTA

Bacterial conjugation and transfer efficiency. Equal amounts (1 ml) of secondary inocula of recipient and donor cells were washed twice with fresh LB to remove residual antibiotics, then mixed and resuspended in 400 μl LB. 35 μl of mixture was dropped on 0.45 μm millipore filter membranes (Sartorius) placed on LB agar plate and incubated at 30 °C for 8 h. For tests of the influence of antibiotics on conjugation, the filter membranes were put on LB agar plates containing sublethal concentrations of indicated antibiotics (1/4 of MIC). Bacteria washed out from the membranes were serially diluted with PBS and plated to determine the bacterial loads. The conjugants were differentiated from other strains based on their antibiotics resistance. The ratios of conjugants counts to donor strains were used to determine the transfer efficiency.

Construction of ∆P::pNQ705K. The kanamycin open reading frame fragment was PCR-amplified from p34S-Km plasmid using the primer pairs KmCS-F (*SalI*)/R (*SalI*) (Table 2), digested with *SalI* and ligated with linearized pNQ705. The resulting plasmid pNQ705K was digested with *XbaI*. The *glms* fragment was amplified from EIB202 genomic DNA using the primer pairs Glms-F/R and cloned into linearized pNQ705K by Gibson assembly [22]. The resulting plasmid pNQ705Km-*glms* were transformed into EIB202 ∆P by conjugation and the targeted EIB202 ∆P::pNQ705K was validated using the primer pair Glms-yz-F/KmCS-R (*SalI*).

Transposon insertion sequencing (TIS) and data analysis. We followed the protocol described previously to create transposon insertion libraries and perform TIS [23]. In brief, overnight cultures of SM10 *λpir*/pMPR (the donor of transposon, Tn) and EIB202/pEIB202 were mixed and incubated for 8 h. Exconjugant cells with the Gm^r^ Tn inserted into the chromosome or plasmid of EIB202 carrying pEIB202 were recovered on a total of 30 plates as Col^r^, Gm^r^, and Str^r^ colonies (~6000 cfu/plate) that were resuspended and collected as the input library. Total plasmid DNA was extracted from a 5 ml aliquot (~1/5) of the input library. Another aliquot of the input library (with the OD_600_ adjusted to match that of an overnight culture recipient) was used as the donor in a second round of conjugation with the new recipient EIB202 ∆p::pNQ705K (Km^r^). Exconjugants were selected as Km^r^, Gm^r^, and Str^r^ colonies; this scheme selects for Tn insertion in pEIB202 (not the chromosome) that are still capable of transmission. About 8 × 10^5^ Km^r^, Gm^r^, and Str^r^ colonies were collected and designated as the output library (*n* = 3). For each library, plasmid DNA (pDNA) from 5 ml bacteria was extracted via the TIANprep Mini Plasmid kit (Tiangen), diluted in 100 μl MiniQ water to a final concentration of 50 ng/μl in the 0.5 ml tube and sonicated by Bioruptor machine (Diagenode) with 30 s ON/90 s OFF for 12 cycles. The sheared DNA length was in the range of 200–600 bp as shown by electrophoresis in a 2% agarose gel. DNA libraries were constructed using the VAHTS Turbo DNA library preparation kit (Vazyme) and sequenced on an Illumina MiSeq platform (Illumina). The sequencing reads were mapped to *E. piscicida* pEIB202 by Bowtie [24]. After mapping, the reads per TA site were tallied and assigned to annotated genes or intergenic regions using previously described scripts [23].

Construction of deletion mutant. In-frame deletion mutants were generated by the *sacB*-based allelic exchange as previously described [25]. Upstream and downstream fragments were generated by PCR with primer pairs P1/P2 and P3/P4 listed in Table 2, respectively. The resulting fragments were cloned into *sacB* suicide vector pDMK using Gibson Assembly and transformed into DH5α *λpir*, clones were validated with the primer pair pDMK-PF/PR. After sequencing, the resulting plasmids were transformed into SM10 *λpir*, and then mated into EIB202 by conjugation. Double crossover processes were selected sequentially on TSA medium containing Col and Km and then on TSA with 12% (*w*/*v*) sucrose to complete homologous recombination. The targeted mutants were confirmed by PCR using the primer pairs in-F/R, out-F/R and *evpP*-F/R, and sequencing of the deleted region.

Determination of minimum inhibitory concentration (MIC). Inocula of WT and ∆*esrB* strains were seeded into LB culture with different amount of antibiotics in 96-well plates and incubated at 30 °C for 24 h.

Determination of pEIB202 copy number. As previous described [26], genomic DNA was extracted from log phase cultures with the gDNA purification kit (Tiangen) and subjected to qPCR in 7500 RealTime PCR System (Applied Biosystems) with FastStart Universal SYBR Green Master (Roche). *virB10* and *gyrB* loci were used for plasmid and chromosome quantification, respectively. A standard plasmid (pD43B) that contains chromosomal (*gyrB*) and plasmid DNA (*virB10*) fragments for qPCR analyses for measurement of pEIB202 copy number was constructed by first amplifying *virB10* and *gyrB* target regions and then inserting them into pDMK by Gibson Assembly. The primers used for these experiments are listed in Table 2.

Total RNA extraction and mRNA enrichment. The WT strain and the *∆esrB* mutant were cultured in LB and DMEM, respectively, at 30 °C without shaking for 24 h. RNA samples were extracted using an RNA isolation kit (Tiangen). DNase I (Promega) and Ribo-Zero-rRNA removal kits for Gram-negative bacteria (Epicentre) were used to remove DNA and rRNA following the manufacturer’s instructions. Prior to reverse transcription, regular PCR was routinely performed using the isolated RNA sample as a template to confirm that there was no DNA contamination. Samples used for RNA-seq were validated using an Agilent 2100 Bioanalyzer (Agilent Technologies), and the final concentration was measured using a Qubit 2.0 Fluorometer (Thermo Fisher).

RNA-seq transcriptome generation and data analysis. First-strand cDNA synthesis from rRNA-depleted samples was carried out using TruSeq RNA sample Prep (Illumina). The cDNA was purified using the RNA Clean and Concentrator-25 kit (Zymo Research). Following second-strand synthesis, the reactions were cleaned up with AMPure XP beads followed by end repair, adenylation of 3′ ends and ligation of adapters. The reaction products were cleaned with AMPure XP beads and treated with uracil-N-glycosylase using the AmpErase kit (Applied Biosystems). Finally, PCR (10 cycles) was used to amplify the library and to enrich the fragments that were ligated to the sequencing adapters. Libraries were sequenced on the HiSeq 2000 platform to yield 100-base-pair end-reads. Adapter sequences and low-quality bases (PHRED quality scores ≤5) were trimmed by the Trimmomatic package using the default parameters, and truncated reads smaller than 35 bp were discarded [27]. Then, the BWA program [28] was used to align the remaining reads to the reference sequences of *E. piscicida* EIB202 (Chr, CP001135.1; plasmid, CP001136.1). The number of reads mapped to each gene was determined by Picard tools (http://broadinstitute.github.io/picard/faq.html) and normalized to the reads per kilobase of genic region per million mapped reads (RPKM) to obtain the relative level of expression. An analysis of variance was performed on the average expression of the three biological replicates to identify genes that showed differential expression under the tested condition (adjusted *P* < 0.05 and 1 fold change). The differential expression analysis was performed using the DEGseq package [29]. The fold changes of genes of interest were validated using qRT-PCR. The RNA-seq datasets have been deposited in the NCBI GenBank under accession number SRP077869.

Zebrafish maintance, LD_50_ and competitive indices analysis. The zebrafish maintenance and challenge were performed as previously described [20]. Healthy zebrafish (*Dario rerio*) of 0.25 ± 0.05 g were raised in reverse osmosis purified water in a flow-through auto-controlled system at 25 °C and acclimatized at least 10 days. The strains of WT, ∆P, and ∆*esrB* were harvested and serially diluted with PBS, respectively. Zebrafish were anesthetized with tricaine methanesulfonate (MS-222, Sigma) at a concentration of 80 mg/L and intramuscularly (i.m.) injected with the strains at doses ranging from 5 × 10 to 5 × 10^5^ CFU/fish. Thirty fish were injected with each dilution and divided randomly into three groups. Fish injected with PBS served as negative controls. The mortalities were recorded over a period of 7 days after infection. The LD_50_ values were calculated via the method described by Fernández et al. [30]. Competitive indices were determined between each strain. The indicated strains were equally mixed and serially diluted with PBS into 1 × 10^3^ CFU/fish. At 72 h post injection, 7 fish were homogenized respectively and plated to determine the bacterial loads. WT (*lacZ*) was differentiated from WT using LBA containing X-Gal (blue and white colonies), and ∆P was differentiated from WT based on Str resistance.

## Results

**Fig. 1:**
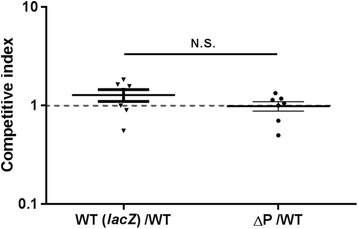
Competitive indices of WT (lacZ)/WT and ∆P/WT in zebrafish at 72 h post-infection. The pEIB202-cured strain (∆P) (Col^r^) was differentiated from WT strains (Str^r^/Col^r^) based on Str and Col resistance. WT (*lacZ*) was differed from WT by blue or white colonies on LB plates supplemented with X-gal and Col. Results were presented as mean ± SD (*n* = 7). N.S., not significant, based on ANOVA followed by Bonferroni’s multiple-comparison posttest

pEIB202 is not required for virulence and colonization of *E. piscicida*. Previous whole genome sequencing indicated that *E. piscicida* EIB202 (previously *E. tarda* EIB202) harbors a multi-drug resistance plasmid pEIB202 [18]. To characterize the roles of pEIB202 on virulence of *E. piscicida*, healthy zebrafish were intramuscularly (i.m.) infected with serial dilutions of bacterial suspensions of WT, ∆P, and ∆*esrB*, respectively. The results indicated that LD_50_ value of EIB202 was 1.5 × 10^2^ CFU/fish during 7 days of observation (Table 3). ∆*esrB* showed significant virulence attenuation with an LD_50_ ~ 1000-fold higher than that of WT, as previously reported [19, 20]. The LD_50_ value of ∆P showed no obvious difference with that of WT. We further analyzed the effect of pEIB202 curing on the colonization of *E. piscicida*. The WT carrying *lacZ*, WT (*lacZ*), was equally mixed with WT and inoculated into the turbot fish. Similarly, ∆P was also used to compete against WT in vivo in fish. After 72 h competition in zebrafish, WT (*lacZ*), WT, and ∆P showed similar colonization capacity (Fig. 1). Collectively, these results indicated that pEIB202 appears to be not required for virulence and in vivo colonization of *E. piscicida*.

**Table 3:** Virulence towards zebrafish (LD_50_) and MIC of each strain

Strains	LD_50_ (CFU/fish)	MIC (μg/ml)			
		Cm	Str	Col	Tet
WT	1.5 × 10^2^	170	>6000	>600	250
∆P	2.0 × 10^2^	1.75	2.3	>600	1.25
Δ*esrB*	1.4 × 10^5^	136	4000	>600	190

**Fig. 2:**
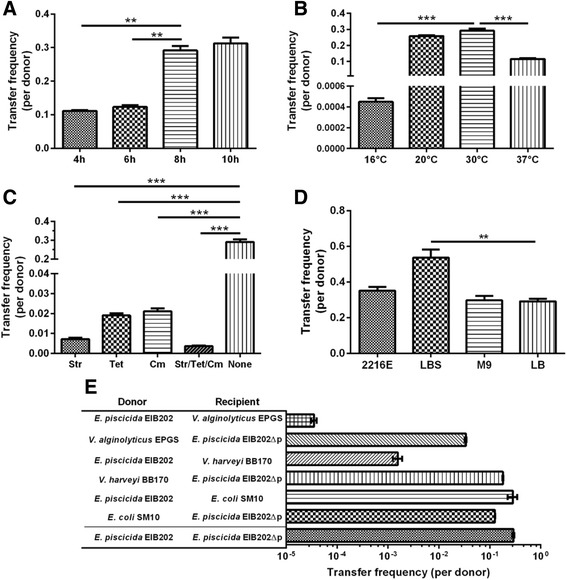
Transfer frequency of pEIB202 under different conditions. The WT strain (donor, Str^r^/Col^r^) carrying pEIB202 plasmid was conjugated with the EIB202 plasmid-cured strain with Km resistant gene inserted in a neutral site of the chromosome (EIB202 ∆P::pNQ705K) (recipient, Km^r^/Col^r^) and the conjugants were selected on Str/Km/Col LB agar plates. **a** Strains were conjugated for different time under 30 °C. **b** Strains were conjugated under different temperatures for 8 h. **c** and **d** Strains were conjugated at different antibiotics at their sublethal concentrations (1/4 of MIC) and culture conditions under 30 °C for 8 h. **e** The transfer frequency between *E. piscicida* and other bacterial species. Results were presented as mean ± SD (*n* = 3). * *P* < 0.01, ** *P* < 0.001, ***P < 0.0001, student’s *t*-test

pEIB202 is self-transmissible under various conditions. Our previous investigation indicated that pEIB202 can be transferred in LB medium [18]. To further analyze the transfer capacity of the plasmid, we generated the strain ∆P::pNQ705K, a pEIB202-cured strain which carries Km resistance gene inserted in a neutral site of the chromosome (Table 1). The conjugation of pEIB202 (carrying Cm resistance) from WT (donor, Str^r^/Col^r^) to ∆P::pNQ705K (recipient, Km^r^/Col^r^) was performed under various conditions and then the conjugants thus could be differentiated from donor and recipient by Km and Str resistance. The transfer efficiency of pEIB202 between WT and the pEIB202-cured strain ∆P::pNQ705K is ~0.1 conjugant/donor after 4 h of conjugation (Fig. 2a). The conjugation efficiency significantly increased to 0.3 conjugant/donor at the time of 8 h incubation (Fig. 2a). The plasmid pEIB202 can transfer through conjugation at the temperature ranging from 16 °C to 37 °C, though 30 °C is the optimum temperature for conjugation (Fig. 2b). We also investigated the conjugation condition with different antibiotics at sublethal concentrations (1/4 of MIC). The plasmid pEIB202 encodes genes against four antibiotics namely chloramphenicol, streptomycin, tetracycline, and sulfonamide [18]. The subinhibitory antibiotic concentrations didn’t cause apparently reduced viability of recipient cells (data not shown). Notably, we found that sublethal of Str, Tet, and Cm significantly inhibited the pEIB202 conjugation transfer, as compared to the standard conjugation operation in laboratory without antibiotics (Fig. 2c). The investigation of conjugation in various media indicated that pEIB202 could transfer in 2216E, M9, and LB with similar efficiency, LBS significantly promotes the conjugation transfer, suggesting that 3% sodium is essential for conjugation processes (Fig. 2d). We also found that pEIB202 could be transferred from EIB202 to enterobacteria (*Escherichia coli*), and also other pathogenic bacteria (*Vibrio alginolyticus* and *V. harveyi*) in marine environments. Otherwise, pEIB202 is also able to transfer back to *E. piscicida* (Fig. 2e). Altogether, these data demonstrated that pEIB202 is able to transfer under various conditions.

Transposon insertion sequencing (TIS) reveals T4SS genes essential for pEIB202 transfer. We used TIS to screen genes in pEIB202 required for conjugation transfer. Suicide plasmid pMPR (Table 1) carrying Himar I mariner transposon was used for high-density mutagenesis of pEIB202. Our rationale is that if a plasmid gene made a positive contribution to (stimulated) conjugation transfer of pEIB202 to the recipient ∆P::pNQ705K, then insertion mutations in that gene would be under-presented in a library after selection for tranconjugants. Conversely, if a gene made a negative contribution to (inhibited) conjugation, then mutations in that gene would be over-presented. We set out to construct a highly saturated Tn-insertion library in WT harboring pEIB202. The set of pEIB202 insertions were subsequently referred to as the input library and used to donate mutated pEIB202 to another recipient ∆P::pNQ705K and the resistant markers on the plasmid and transposon were used for selection of exconjugants. The set of pEIB202 insertion mutants in the ∆P::pNQ705K exconjugants is referred to below as the output library. We compared the distribution of transposon insertion sites in the input and output libraries and identify pEIB202 genes affecting plasmid transfer with the previously described analysis pipeline [23, 31]. Insertions in genes in pEIB202 that are underrepresented or absent in the output library (relative to their abundance in the input library) likely correspond to loci that facilitate pEIB202 transfer, and over-represented in the output library likely negatively affect the plasmid transfer.

**Fig. 3:**
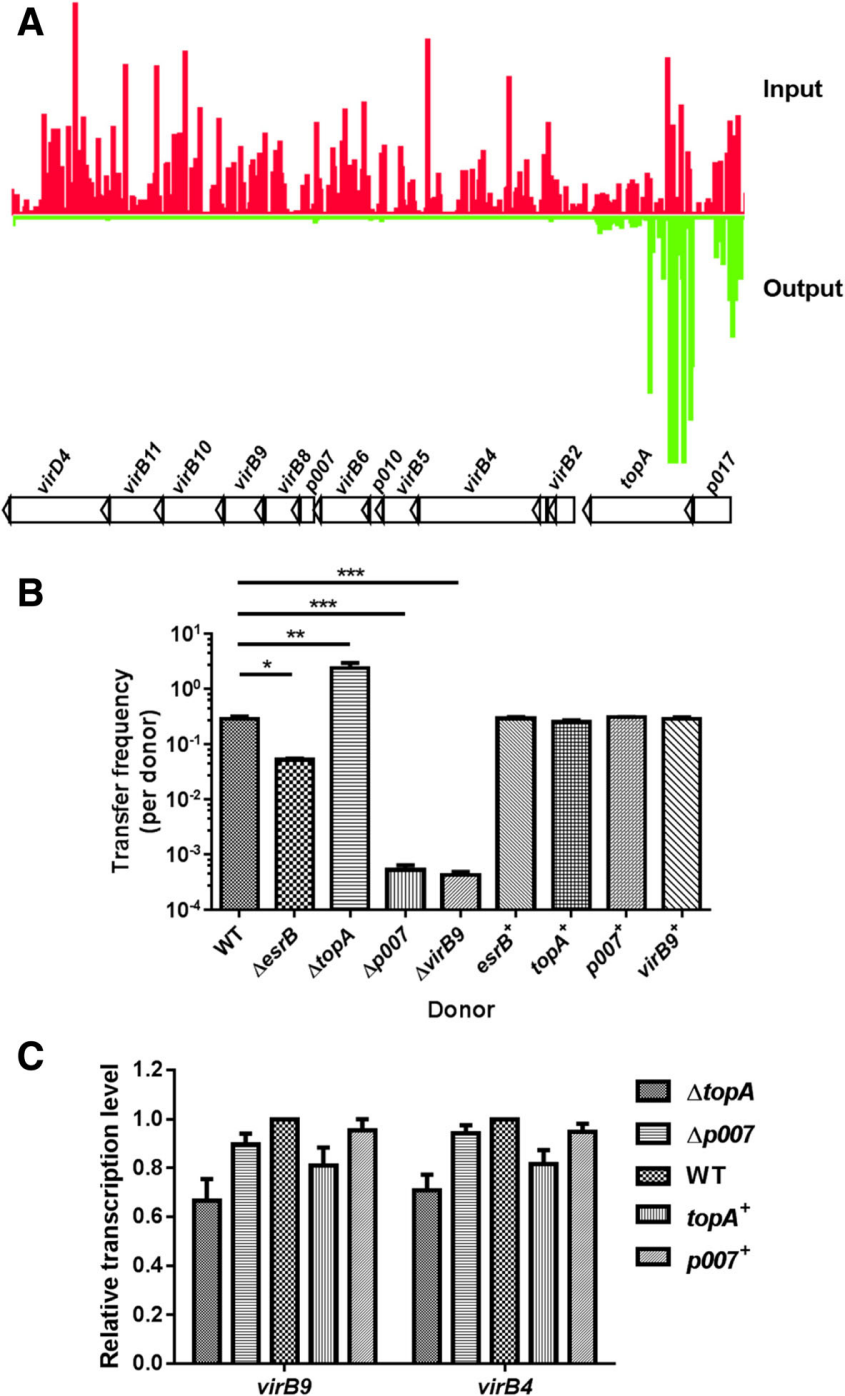
TIS identification and verification of genes associated with pEIB202 transfer. **a** Artemis screenshot of abundance of reads in *topA*, *p007* and T4SS genes in input (*red*) and output (*green*) samples. The height of the *red* and *green* bars correlates with the number of reads. **b** The transfer frequency of pEIB202 from each strain to ∆P::pNQ705K. Strains were conjugated at 30 °C for 8 h. **c** qRT-PCR analysis of the expression of T4SS genes in each strain grown in LB. * *P* < 0.01, ** *P* < 0.001, ***P < 0.0001, student’s *t*-test

The input library included approximately 100,000 reads that mapped to pEIB202 and covered 88.1% of the plasmid TA sites, suggesting that the library is sufficiently saturated to identify genes essential for pEIB202 transfer. In the input library, the Tn insertions were fairly evenly distributed around pEIB202. The three output libraries contained 109,678 reads (covered 62.2% of TA), 110,322 reads (covered 62.4% of TA) and, 97,595 reads (62.1% of TA), respectively. Using the HMM-based analytic pipeline [23], we could profile the gene loci that possibly affect the replication, maintenance, conjugation transfer, or selection processes of pEIB202 in *E. piscicida*. Some genes, including the *tra*, *rep*, and antibiotics resistance genes, showed significantly increased or decreased output reads but with lower output/input ratios (< 4-fold change), suggesting that they might contribute to the maintenance and replication or the selection process after conjugation (data not shown). Their exact roles could not be assigned for the above-mentioned complex processes and for the possibility of redundancy for each of the gene from the chromosome or plasmid encoded genes. We identified 20 genes with output/input ratios higher than 4-fold which might significantly affect the conjugation transfer of pEIB202 (Table 4). Notably, all of the ten T4SS VirB proteins were identified as genes essential for pEIB202 transfer with a significantly (*P* < 0.001) decreased (by 2–3 log) output. The gene *topA*, encoding a DNA topoisomerase I, was the only candidate that inhibiting pEIB202 transfer (Table 4) (Fig. 3a). Insertions in the TA loci in this gene caused 4.30-fold increase (*P* < 0.001) in the output reads. All the regions related to the above-described 20 genes shared averaged input reads, suggesting that these genes were not associated with the maintenance or replication, but conjugation transfer in *E. piscicida.*

**Table 4:** Genes identified by TIS in pEIB202 that affects maintenance or conjugation of pEIB202

Loci	Gene	Product	Output/input ^a^	*P*-value
ETAE_p001	*virD4*	T4SS component VirD4	0.015	0.000548801
ETAE_p002	*virB11*	T4SS component VirB11	0.0058	0.000531746
ETAE_p003	*virB10*	T4SS component VirB10	0.00099	0.000531259
ETAE_p004		hypothetical protein	0.0022	0.000517762
ETAE_p005	*virB9*	T4SS component VirB9	0.0039	0.000520783
ETAE_p006	*virB8*	T4SS component VirB8	0.0014	0.000530141
ETAE_p007		putative lipoprotein	0.0034	0.000493853
ETAE_p008		hypothetical protein	0.10	0.0003746
ETAE_p009	*virB6*	T4SS component VirB6	0.011	0.000544597
ETAE_p010		putative lipoprotein	0.18	1.61153E-05
ETAE_p011	*virB5*	T4SS component VirB5	0.0027	0.000535705
ETAE_p012		hypothetical protein	0.026	0.0003982
ETAE_p013	*virB4*	T4SS component VirB4	0.0086	0.000541165
ETAE_p014	*virB2*	T4SS component VirB2	0.019	0.000514194
ETAE_p015		hypothetical protein	0.073	9.96994E-06
ETAE_p016	*topA*	DNA topoisomerase	4.30	0.000340276
ETAE_p026		transcriptional repressor protein	0.020	6.77947E-06
ETAE_p047	*mobC*	putative mobilisation protein	0.13	0.000639706
ETAE_p048	*virD2*	VirD2 component	0.028	0.000545018
ETAE_p053	*traC*	DNA primase TraC4	0.15	2.38855E-05

^a^The data indicate that the read number of a specific gene in output is significantly under-represented or over-represented as compared to that in input with a cutoff of less than 0.25 or higher than 4-fold and *p* < 0.001

In order to further verify the TIS identified genes as the regulators or modulators controlling pEIB202 transfer, we constructed in-frame deletion mutants and corresponding complement strains for *topA*, *p007*, and *virB9* and determined the transfer efficiency of the mutated pEIB202. The mutant with a deletion in *virB9*, encoding the channel component protein essential for T4SS function [32], showed significantly reduced transfer frequency (Fig. 3b), suggesting that, as expected, the proper function of T4SS is required for pEIB202 horizontal transfer. Putative lipoprotein p007 was also proved to be important for pEIB202 transfer (Fig. 3b). The plasmid without gene *topA* has a significantly higher transfer frequency in comparison to that of the WT (Fig. 3b). The transfer frequencies of their complement strains were recovered to the WT level (Fig. 3b).

It was intriguingly to find that *p007* and *topA* are involved in the conjugation transfer. We further asked whether deletion of these genes in pEIB202 would affect the expression of *virB* genes, which subsequently modulates the T4SS function and activities to influence plasmid conjugation processes. We carried out qRT-PCR to test the expression of *virB9* and *virB4*, encoding two core proteins for T4SS function, in WT, Δ*p007* and Δ*topA* strains as well as their complement strains. The expression levels of *virB* genes were somewhat decreased when *topA* was absent; while that of Δ*p007* were similar to that of the WT (Fig. 3c). In addition, the relative transcription levels of *virB9* and *virB4* were not changed much when *topA* or *p007* genes were back complemented into their respective deletion mutants, suggesting that the alterations of conjugation capacities in Δ*p007* and Δ*topA* might not be associated with the modulation of T4SS expression; Taken together, these data demonstrated that *virB* genes, *p007* and *topA* are essential for the pEIB202 transfer.

**Fig. 4:**
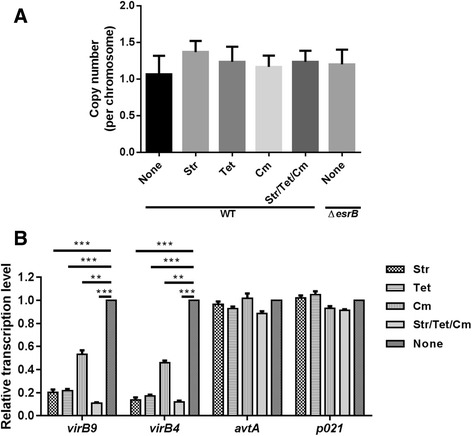
Antibiotics inhibit the expression of T4SS but does not affect the copy number of pEIB202. **a** Copy number of pEIB202 in WT grown with or without antibiotics at their sublethal concentrations or in ∆*esrB* measured by qPCR. **b** qRT-PCR analysis of the expression of T4SS genes in WT grown with or without antibiotics at their sublethal concentrations (1/4 of MIC). * *P* < 0.01, ** *P* < 0.001, ***P < 0.0001, student’s *t*-test

Antibiotics modulate T4SS gene expression in pEIB202. We were intrigued that how antibiotics could modulate the pEIB202 conjugation frequency in *E. piscicida* as shown in Fig. 2c. We focused on the influences of antibiotics on plasmid conjugation as a whole process but did not consider the influence of antibiotics on the recipients’ activities. We first asked whether the copy number of pEIB202 would be significantly changed with the addition of sublethal antibiotics (1/4 of MIC) Str, Tet, and Cm. The data indicated that the copy number of pEIB202 was 1.2–1.5 per cell and appeared not to be significantly affected by the presence of the sublethal antibiotics Str, Tet, and Cm during conjugation (Fig. 4a). In the TIS data, the 6 antibiotics resistance genes shared the averaged input reads and showed no variations in the output reads (Table 4), suggesting that the carrying of antibiotics resistance genes might not affect the pEIB202 transmission. qRT-PCR was further performed to analyze the expression of *virB9* and *virB4* in WT grown with or without sublethal concentrations of antibiotics Str, Tet, and Cm. The transcriptional level of T4SS genes (*virB4* and *virB9*) was significantly reduced due to the addition of antibiotics as compared to that without antibiotics and the expression of other unrelated genes on chromosome or the plasmid (Fig. 4b), suggesting an inhibitory effect of these antibiotics on the expression T4SS in pEIB202 at sublethal concentrations.

**Fig. 5:**
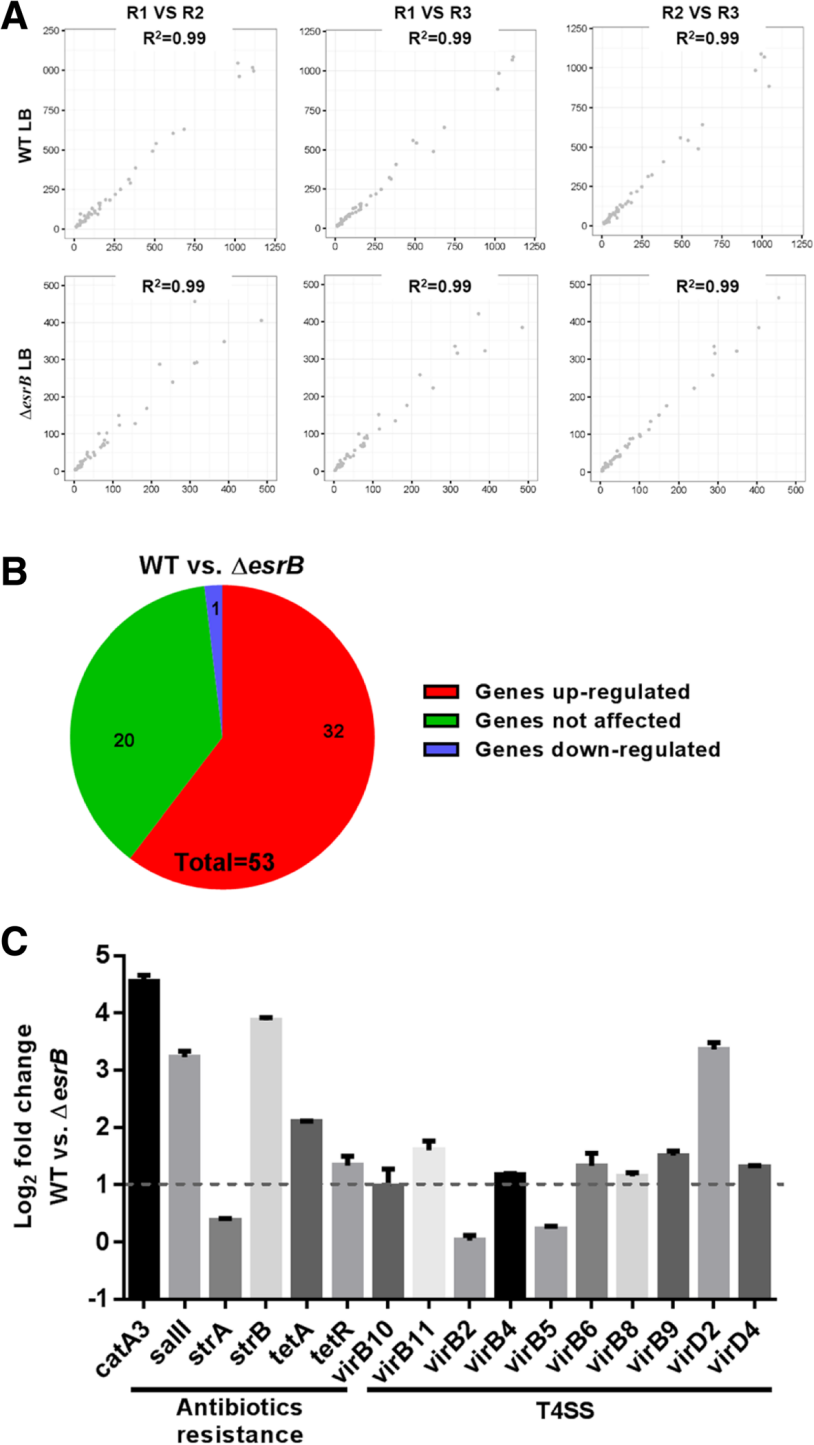
RNA-seq analysis of differential plasmid gene expression of WT and ∆*esrB* cultured in LB (*n* = 3) based on the normalized transcript levels. **a** Scatter plots of RPKM values per ORF in pEIB202 in biological replicates R1, R2, and R3. **b** Numbers of differentially transcribed plasmid genes (fold change >2). **c** Relative expression of antibiotics resistance and T4SS genes of pEIB202 that are differentially expressed in WT versus ∆*esrB*

EsrB activates the expression of T4SS and antibiotic resistance genes. The response regulator EsrB encoded in the horizontal acquired T3SS gene cluster is essential for the bacterial virulence (Table 3) [20]. Intriguingly, our results also showed that absence of *esrB* in the chromosome significantly decreased the conjugation frequency in *E. piscicida* (Fig. 3b). The copy number of pEIB202 was kept no change when *esrB* was absent as compared to the WT (Fig. 4a). In order to interrogate this, we performed RNA-seq analysis with EIB202 and ∆*esrB* RNA isolated from LB (*n* = 3) to compare their differential gene expression. RNA-Seq analysis was performed in triplicate and the reproducibility of the biological repeats was extremely high (a mean R^2^–0.99, Fig. 5a). A total of 33 (62%) plasmid genes were up-regulated by at least 2-fold in WT, when WT and ∆*esrB* transcriptoms were compared (Fig. 5b). These data suggested that EsrB also serves as an activator of plasmid gene expression in *E. piscicida*. Notably, among all the 6 antibiotic resistance genes and 10 T4SS genes in pEIB202 nearly all antibiotics resistance (except *strA*) and T4SS genes (except *virB2* and *virB5*) were markedly activated by EsrB (Fig. 5c). We further compared the MIC of Cm, Str, Col, and Tet for WT and Δ*esrB*. The data indicated that, as compared to that of WT, Δ*esrB* showed significantly decreased resistance towards Cm, Str, and Tet, but remain similar Col resistance (Table 3), which is encoded on the chromosome. These data were well supporting the finding that EsrB activates the antibiotics gene expression in the plasmid. Collectively, our data demonstrated that EsrB is essential for the conjugation transfer of pEIB202, probably through regulating the expression of T4SS in LB.

## Discussion

Bacterial antibiotics resistance has increasingly become a globally recognized concern. The capacities to exchange antibiotics resistance determinants conferred by horizontal gene transfer (HGT) have greatly intensified the issue. As a leading fish pathogen, *E. piscicida* harbors an array of antibiotics-resistance determinants in both of its chromosome and plasmids and well prepares for the antibiotics cocktail that might be present in the aquaculture environments [18]. Furthermore, *E. piscicida* is an intracellular pathogen residing various cell types including phagocytes and epithelial cells, which may help the bacterium to escape the antibiotics attack and persist in the ecosystems. The way antibiotics affect the conjugation process and interact with the genetic basis of conjugation is still confusing. Here we used the multi-drug resistance plasmid pEIB202 from a highly virulent *E. piscicida* strain EIB202 [33] to address this and our findings indicated that antibiotics or their mixture modulate the conjugation efficiencies. The T4SS genes in pEIB202 are required for the conjugation process. Antibiotics appear to modulate conjugation frequencies by repressing T4SS gene expression. Furthermore, the response regulator EsrB encoded on the chromosome is also essential to facilitate the conjugation by up-regulating the T4SS genes. Investigation of the gene transfer mediated by the plasmid conjugation in the growth conditions mimic of aquaculture farms will improve the understanding of requirements for plasmid conjugation processes and help evaluation of antibiotics treatment in the industries.

Genetic analysis of pEIB202 indicated that it belongs to IncP plasmid and is capable of replication and stable inheritance in a wide variety of gram-negative bacteria [18]. As compared to other reported plasmids carried by *Edwardsiella* bacterium, such as pCK41 (70 kb) from *E. piscicida* [34], p080813–1 (127 kb) from *E. anguillarum* [35], and pEI1/2 (3.9 kb and 4.8 kb) and similar plasmids from *E. ictaluri* [36, 37], pEIB202 (43 kb) found in *E. piscicida* strain EIB202 is medium sized. In some *Edwardsiella* strains, there are no plasmids. While some of the plasmids, i.e. pEI1/2 encoding T3SS genes, are highly associated to the bacterial pathogenesis [34, 36], many of the plasmids including pEIB202 in the bacterium bear multi-drug resistances and seems to mainly contribute to their survival in the antibiotics selection, while not the pathogenesis (Fig. 1 and Table 3). It is also notable that chloramphenicol resistance plasmids from *E. piscicida* were increasingly reported in recent years in the aquaculture farms [18, 35, 38, 39]. Given that the presence of *catA3* gene and the neighboring complex transposon ISSf1 containing IS4 family transposase, as well as the extremely low G + C content of this region (37.4%), as compared to average G + C content of the plasmid (57.3%) or of the genome (59.7%), the chloramphenicol resistance might emerge from a recent acquisition event through HGT in pEIB202.

To make the situations severe, pEIB202 carries a set of T4SS which is believed and now proved by our experiment here to potently facilitate the conjugation and dissemination of the antibiotics-resistance genes in the environment. This should not only make the *E. piscicida* EIB202 and other strains recalcitrant in aquaculture farms, but may also facilitate the HGT of pathogenic islands and their evolution for the emerging of other highly pathogenic strains like *E. anguillarum* [35, 40]. Indeed, pEIB202 can transfer through conjugation at various conditions with high frequencies between different bacterial species (Fig. 2). With this in mind, we developed our recent licensed live attenuated vaccine WED from EIB202 by curing of pEIB202 for biosafety concerns [41]. The plasmid pEIB202 encodes an incomplete set of components of T4SS namely VirB2, −B4, −B5, −B6, −B8, −B9, −B10, −B11, −D2, and-D4 without –B1, −B3, −B7 [18]. Structural and biochemical analysis of T4SS indicated that the inner membrane protein VirB3 is essential for assembly of the T4SS translocon and the interaction of VirB3 with VirB7 and VirB8 is crucial for the stabilization [32, 42]. We identified all these rest of 10 proteins can promote the transfer of pEIB202 (Fig. 3a, Table 4). These data suggest that the chromosome- encoded proteins might be involved in rescuing the functions of VirB3 or other two VirB proteins when they are missing. The conjugation frequency of pEIB202 decreased significantly in ∆*virB4* and ∆*virB9* (Fig. 3b). All these results provided evidence that this incomplete T4SS is functional and plays an important role in the horizontal transfer of pEIB202. Whether and how the protein substrates could be transferred by the T4SS in pEIB202 should be further illuminated in the future.

Besides established T4SS genes, other genes were also revealed to be essential for pEIB202 conjugation transfer. The genes encoding lipoproteins p007, p010 and two hypothetical proteins and neighboring the T4SS genes were identified to be essential for pEIB202 transfer (Fig. 3b, Table 4). LpqM, a mycobacterial lipoprotein-metalloproteinase, was also required for conjugal DNA transfer, while the requirement of LpqM for conjugation is specific to donor strain but not recipient [43]. Lipoproteins constitute a significant proportion of the cell wall, and may play certain roles in the assembly or function of T4SS and further plasmid transfer. It is anticipated that *topA* influences conjugation processes in this study. The gene *topA* encodes topoisomerase I which is an enzyme essential for relaxation of DNA during a number of critical cellular process [44]. During the process of the plasmid horizontal transfer, topoisomerase I works as an inhibitor, as it breaks and reseals phosphodiester bonds to relax the supercoiled DNA. The role of *p026* encoding a transcriptional repressor in the conjugation transfer remains to be investigated (Table 4). Among all the established *mob*/*tra* proteins [45, 46], only *p047* for MobC and *p053* for TraC were observed to be essential for conjugation transfer, though with less effect as compared to T4SS genes (Table 4). How T4SS facilitate the *mob*/*tra* mediated conjugation processes and their interaction should be further investigated. Besides the DNA regions encoding specific proteins, some intergenic regions seem also to be required for plasmid maintenance or conjugation transfer. For example, there was no insertion in the regions ETAE_p015--ETAE_p016 and ETAE_p033--ETAE_p034 in the input libraries, suggesting their essentiality for the plasmid maintenance or replication in *E. pisicicida* (data not shown). While for the region ETAE_p004-- ETAE_ p005 and ETAE_p007-- ETAE_ p008, the no insertion may be resulted from the polar effect for the T4SS gene expression or they are the DNA regions for origin of transfer (*oriT*) per se (data not shown). In addition to the above-mentioned plasmid genes or DNA regions, EsrB was intriguingly identified to control the plasmid conjugation transfer (Fig. 3b). EsrA-EsrB is the two-component system essential for intracellular survival in *E. piscicida* [47], and plays a key role in regulating ~400 genes’ expression (personal communication, Wang QY). However, no plasmid gene controlled by EsrA-EsrB was reported. Here, we showed that over half of the pEIB202 genes were induced by EsrB, which facilitate the conjugation in part by upregulating the T4SS genes (Fig. 5). It should be noted that although EsrB expression is significantly higher in the nutrient defined DMEM medium than in LB, but high level of EsrB expression could also be detected in cells grown in LB [48]. We only tested the modulation of pEIB202 genes’ expression by EsrB when the cells are growing in LB, the same condition we used for conjugation experiment.

It has been generally assumed that antibiotics promote conjugation [49], while antibiotics of sublethal concentrations can increase the conjugation rate by either activating the excision of transferrable genes from the host chromosome or by inducing the expression of conjugation machinery [50–52]. There were also conflicting reports regarding whether or not antibiotics promote conjugation. Recent study identified that antibiotics of sublethal concentrations do not significantly increase the conjugation efficiency, but dictate antibiotic-mediated selection, which can both promote and suppress conjugation dynamics [1]. Here, we found that Str, Tet and Cm and their mixture at their sublethal concentrations inhibit the conjugation transfer probably mainly due to the repression of T4SS genes (Fig. 2c and 4b), although the influence of the antibiotics on the recipients’ activities should be further considered in the future.

## Conclusion

Our data demonstrated that T4SS is essential for the conjugation transfer of multiple-antibiotic resistant pEIB202 and suggested that antibiotics could affect conjugation processes through modulating T4SS. The results also indicated that the genetic elements in chromosome, especially the horizontally acquired response regulator EsrB could also affect the plasmid transfer through modulating the expression of T4SS. Our results will improve the understanding of the mechanism of plasmid conjugation processes that facilitate dissemination of antibiotic resistance especially in aquaculture industries.

## Abbreviations

ABR: Antibiotic resistance
Carb: Carbenicillin
Cm: Chloramphenicol
Col: Polymyxin B
Gm: Gentamicin
HGT: Horizontal gene transfer
i.m: Intramuscularly
Km: Kanamycin
LB: Luria broth
MIC: Minimum inhibition concentration
pDNA: Plasmid DNA
qRT-PCR: Quantitative real-time PCR
R plasmid: Resistance-encoding plasmid
RPKM: Reads per kilobase of genic region per million mapped reads
Str: Streptomycin
T3SS: Type III secretion systems
T4SS: Type IV secretion system
T6SS: Type VI secretion systems
Tet: Tetracycline
TIS: Transposon insertion sequencing
TSB: Tryptic soy broth

## References

[1] Lopatkin AJ, Huang S, Smith RP, Srimani JK, Sysoeva TA, Bewick S, Karig DK, You L (2016). Antibiotics as a selective driver for conjugation dynamics. Nat Microbiol.

[2] Sorensen SJ, Bailey M, Hansen LH, Kroer N, Wuertz S (2005). Studying plasmid horizontal transfer in situ: a critical review. Nat Rev Microbiol.

[3] Maiden MC (1998). Horizontal genetic exchange, evolution, and spread of antibiotic resistance in bacteria. Clin Infect Dis.

[4] Juhas M (2015). Horizontal gene transfer in human pathogens. Crit Rev Microbiol.

[5] Turner SL, Bailey MJ, Lilley AK, Thomas CM (2002). Ecological and molecular maintenance strategies of mobile genetic elements. FEMS Microbiol Ecol.

[6] van Elsas JD, Bailey MJ (2002). The ecology of transfer of mobile genetic elements. FEMS Microbiol Ecol.

[7] Bruto M, James A, Petton B, Labreuche Y, Chenivesse S, Alunno-Bruscia M, Polz MF, Le Roux F (2016). *Vibrio crassostreae*, a benign oyster colonizer turned into a pathogen after plasmid acquisition. ISME J.

[8] Rhodes G, Huys G, Swings J, McGann P, Hiney M, Smith P, Pickup RW (2000). Distribution of oxytetracycline resistance plasmids between aeromonads in hospital and aquaculture environments: implication of Tn1721 in dissemination of the tetracycline resistance determinant tet a. Appl Environ Microbiol.

[9] Popa O, Dagan T (2011). Trends and barriers to lateral gene transfer in prokaryotes. Curr Opin Microbiol.

[10] Fronzes R, Christie PJ, Waksman G (2009). The structural biology of type IV secretion systems. Nat Rev Microbiol.

[11] Cascales E, Christie PJ (2003). The versatile bacterial type IV secretion systems. Nat Rev Microbiol.

[12] Backert S, Meyer TF (2006). Type IV secretion systems and their effectors in bacterial pathogenesis. Curr Opin Microbiol.

[13] Alvarez-Martinez CE, Christie PJ (2009). Biological diversity of prokaryotic type IV secretion systems. Microbiol Mol Biol Rev.

[14] Park SB, Aoki T, Jung TS (2012). Pathogenesis of and strategies for preventing *Edwardsiella tarda* infection in fish. Vet Res.

[15] Tan YP, Zheng J, Tung SL, Rosenshine I, Leung KY (2005). Role of type III secretion in *Edwardsiella tarda* virulence. Microbiology.

[16] Okuda J, Kiriyama M, Suzaki E, Kataoka K, Nishibuchi M, Nakai T (2009). Characterization of proteins secreted from a type III secretion system of *Edwardsiella tarda* and their roles in macrophage infection. Dis Aquat Org.

[17] Hou M, Chen R, Yang D, Nunez G, Wang Z, Wang Q, Zhang Y, Liu Q (2017). Identification and functional characterization of EseH, a new effector of the type III secretion system of *Edwardsiella piscicida*. Cell Microbiol.

[18] Wang Q, Yang M, Xiao J, Wu H, Wang X, Lv Y, Xu L, Zheng H, Wang S, Zhao G (2009). Genome sequence of the versatile fish pathogen *Edwardsiella tarda* provides insights into its adaptation to broad host ranges and intracellular niches. PLoS One.

[19] Yang M, Lv Y, Xiao J, Wu H, Zheng H, Liu Q, Zhang Y, Wang Q (2012). *Edwardsiella* comparative phylogenomics reveal the new intra/inter-species taxonomic relationships, virulence evolution and niche adaptation mechanisms. PLoS One.

[20] Lv Y, Xiao J, Liu Q, Wu H, Zhang Y, Wang Q (2012). Systematic mutation analysis of two-component signal transduction systems reveals EsrA-EsrB and PhoP-PhoQ as the major virulence regulators in *Edwardsiella tarda*. Vet Microbiol.

[21] Livak KJ, Schmittgen TD (2001). Analysis of relative gene expression data using real-time quantitative PCR and the 2(-Delta Delta C (T)) method. Methods.

[22] Gibson DG, Young L, Chuang RY, Venter JC, Hutchison CR, Smith HO (2009). Enzymatic assembly of DNA molecules up to several hundred kilobases. Nat Methods.

[23] Chao MC, Pritchard JR, Zhang YJ, Rubin EJ, Livny J, Davis BM, Waldor MK (2013). High-resolution definition of the *Vibrio cholerae* essential gene set with hidden Markov model-based analyses of transposon-insertion sequencing data. Nucleic Acids Res.

[24] Langmead B, Trapnell C, Pop M, Salzberg SL (2009). Ultrafast and memory-efficient alignment of short DNA sequences to the human genome. Genome Biol.

[25] Xiao J, Wang Q, Liu Q, Xu L, Wang X, Wu H, Zhang Y (2009). Characterization of *Edwardsiella tarda rpoS*. effect on serum resistance, chondroitinase activity, biofilm formation, and autoinducer synthetases expression. Appl Microbiol Biotechnol.

[26] Yamaichi Y, Chao MC, Sasabe J, Clark L, Davis BM, Yamamoto N, Mori H, Kurokawa K, Waldor MK (2015). High-resolution genetic analysis of the requirements for horizontal transmission of the ESBL plasmid from *Escherichia coli* O104:H4. Nucleic Acids Res.

[27] Bolger AM, Lohse M, Usadel B (2014). Trimmomatic. a flexible trimmer for Illumina sequence data. Bioinformatics.

[28] Li H, Durbin R (2009). Fast and accurate short read alignment with burrows-wheeler transform. Bioinformatics.

[29] Anders S, Huber W (2010). Differential expression analysis for sequence count data. Genome Biol.

[30] Fernandez AI, Perez MJ, Rodriguez LA, Nieto TP (1995). Surface phenotypic characteristics and virulence of Spanish isolates of *Aeromonas salmonicida* after passage through fish. Appl Environ Microbiol.

[31] Pritchard JR, Chao MC, Abel S, Davis BM, Baranowski C, Zhang YJ, Rubin EJ, Waldor MK (2014). ARTIST: high-resolution genome-wide assessment of fitness using transposon-insertion sequencing. PLoS Genet.

[32] Low HH, Gubellini F, Rivera-Calzada A, Braun N, Connery S, Dujeancourt A, Lu F, Redzej A, Fronzes R, Orlova EV (2014). Structure of a type IV secretion system. Nature.

[33] Xiao J, Wang Q, Liu Q, Wang X, Liu H, Zhang Y (2008). Isolation and identification of fish pathogen *Edwardsiella tarda* from mariculture in China. Aquac Res.

[34] Yu JE, Cho MY, Kim JW, Kang HY (2012). Large antibiotic-resistance plasmid of *Edwardsiella tarda* contributes to virulence in fish. Microb Pathog.

[35] Shao S, Lai Q, Liu Q, Wu H, Xiao J, Shao Z, Wang Q, Zhang Y (2015). Phylogenomics characterization of a highly virulent *Edwardsiella* strain ET080813(T) encoding two distinct T3SS and three T6SS gene clusters: propose a novel species as *Edwardsiella anguillarum* sp. nov. Syst Appl Microbiol.

[36] Zhao LJ, Lu JF, Nie P, Li AH, Xiong BX, Xie HX (2013). Roles of plasmid-encoded proteins, EseH, EseI and EscD in invasion, replication and virulence of *Edwardsiella ictaluri*. Vet Microbiol.

[37] Fernandez DH, Pittman-Cooley L, Thune RL (2001). Sequencing and analysis of the *Edwardsiella ictaluri* plasmids. Plasmid.

[38] Welch TJ, Evenhuis J, White DG, McDermott PF, Harbottle H, Miller RA, Griffin M, Wise D (2009). IncA/C plasmid-mediated florfenicol resistance in the catfish pathogen *Edwardsiella ictaluri*. Antimicrob Agents Chemother.

[39] Sun K, Wang H, Zhang M, Xiao Z, Sun L (2009). Genetic mechanisms of multi-antimicrobial resistance in a pathogenic *Edwardsiella tarda* strain. Aquaculture.

[40] Nakamura Y, Takano T, Yasuike M, Sakai T, Matsuyama T, Sano M (2013). Comparative genomics reveals that a fish pathogenic bacterium *Edwardsiella tarda* has acquired the locus of enterocyte effacement (LEE) through horizontal gene transfer. BMC Genomics.

[41] Xiao J, Chen T, Liu B, Yang W, Wang Q, Qu J, Zhang Y (2013). *Edwardsiella tarda* mutant disrupted in type III secretion system and chorismic acid synthesis and cured of a plasmid as a live attenuated vaccine in turbot. Fish Shellfish Immunol.

[42] den Hartigh AB, Rolan HG, de Jong MF, Tsolis RM (2008). VirB3 to VirB6 and VirB8 to VirB11, but not VirB7, are essential for mediating persistence of *Brucella* in the reticuloendothelial system. J Bacteriol.

[43] Nguyen KT, Piastro K, Derbyshire KM (2009). LpqM, a mycobacterial lipoprotein-metalloproteinase, is required for conjugal DNA transfer in *Mycobacterium smegmatis*. J Bacteriol.

[44] Wang JC (2002). Cellular roles of DNA topoisomerases: a molecular perspective. Nat Rev Mol Cell Biol.

[45] Meyer R (2000). Identification of the mob genes of plasmid pSC101 and characterization of a hybrid pSC101-R1162 system for conjugal mobilization. J Bacteriol.

[46] Farrand SK, Hwang I, Cook DM (1996). The tra region of the nopaline-type Ti plasmid is a chimera with elements related to the transfer systems of RSF1010, RP4, and F. J Bacteriol.

[47] Chakraborty S, Sivaraman J, Leung KY, Mok YK (2011). Two-component PhoB-PhoR regulatory system and ferric uptake regulator sense phosphate and iron to control virulence genes in type III and VI secretion systems of *Edwardsiella tarda*. J Biol Chem.

[48] Yin K, Wang Q, Xiao J, Zhang Y (2017). Comparative proteomic analysis unravels a role for EsrB in the regulation of reactive oxygen species stress responses in *Edwardsiella piscicida*. FEMS Microbiol Lett.

[49] Andersson DI, Hughes D (2014). Microbiological effects of sublethal levels of antibiotics. Nat Rev Microbiol.

[50] Stevens AM, Shoemaker NB, Li LY, Salyers AA (1993). Tetracycline regulation of genes on Bacteroides conjugative transposons. J Bacteriol.

[51] Whittle G, Shoemaker NB, Salyers AA (2002). Characterization of genes involved in modulation of conjugal transfer of the Bacteroides conjugative transposon CTnDOT. J Bacteriol.

[52] Beaber JW, Hochhut B, Waldor MK (2004). SOS response promotes horizontal dissemination of antibiotic resistance genes. Nature.

[53] Milton DL, Norqvist A, Wolf-Watz H (1992). Cloning of a metalloprotease gene involved in the virulence mechanism of *Vibrio anguillarum*. J Bacteriol.

[54] Wang SY, Lauritz J, Jass J, Milton DL (2002). A ToxR homolog from *Vibrio anguillarum* serotype O1 regulates its own production, bile resistance, and biofilm formation. J Bacteriol.

[55] Dennis JJ, Zylstra GJ (1998). Plasposons: modular self-cloning minitransposon derivatives for rapid genetic analysis of gram-negative bacterial genomes. Appl Environ Microbiol.

